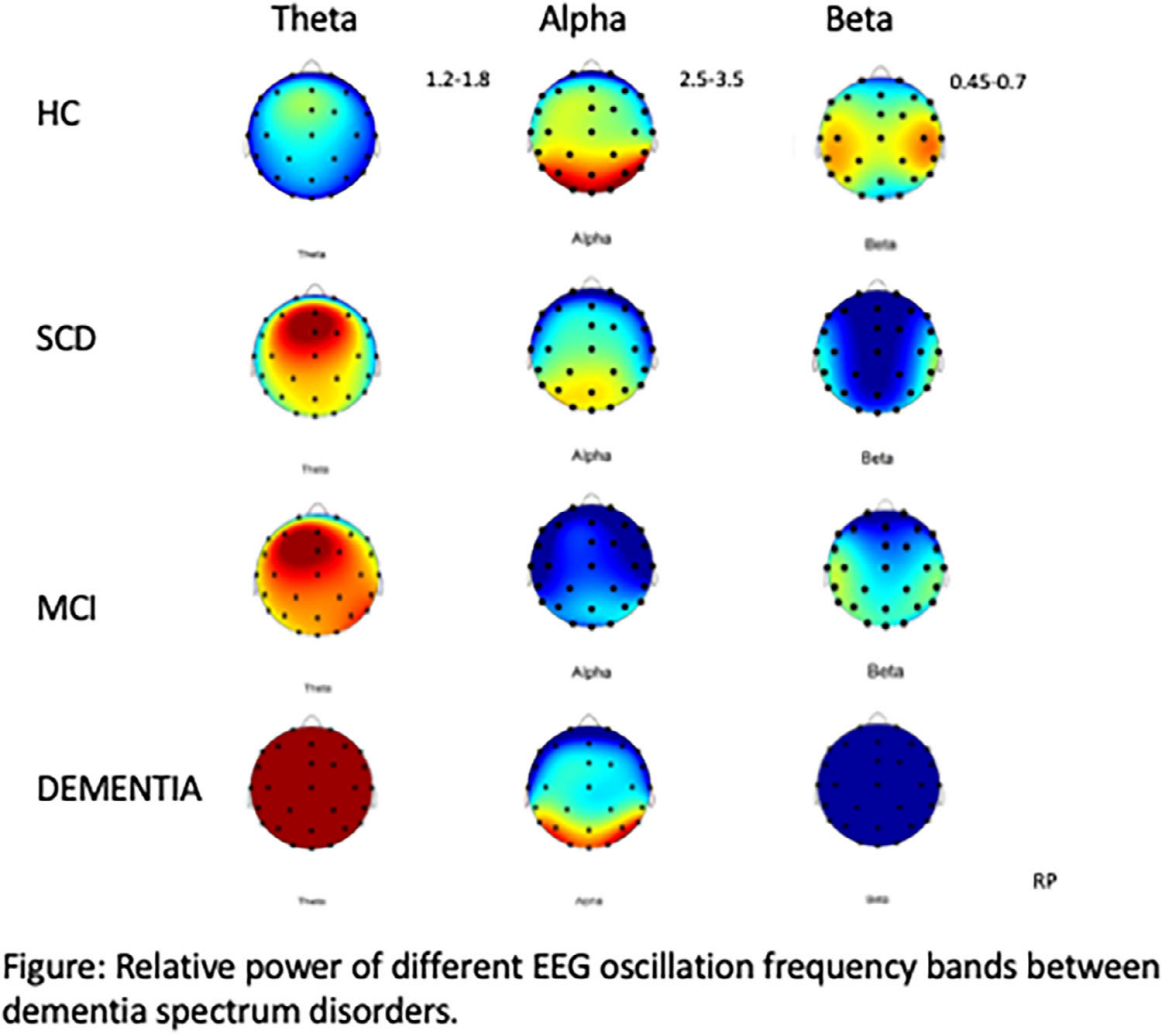# Impaired Complex Activities of Daily Living Correlated with Altered EEG Oscillations in the Dementia Spectrum Disorder

**DOI:** 10.1002/alz.091649

**Published:** 2025-01-09

**Authors:** Poyu Chen, Chin‐Yi Wu, Yu‐hua Huang, Jung‐Lung Hsu, I‐Ching Chuang, Sietske A.M Sikkes

**Affiliations:** ^1^ Chang Gung University, Taipei Taiwan; ^2^ Chang Gung University, Taoyuan, NA Taiwan; ^3^ Chang Gung Memorial Hospital, Taoyuan, NA Taiwan; ^4^ TuCheng Hospital, New Taipei City Taiwan; ^5^ Alzheimer Center Amsterdam, Neurology, Vrije Universiteit Amsterdam, Amsterdam UMC location VUmc, Amsterdam Netherlands

## Abstract

**Background:**

Literature on biomarker studies suggests that pathological changes begin approximately 10 to 20 years before the first cognitive symptom appears in dementia populations. It is an emerging era for developing methods to detect the early signs of progressive cognitive decline. Recently, the instrumental activity of living (IADL) capacity has been regarded as a functional biomarker to predict the progression from MCI to dementia. EEG, combined with cognitive performance, such as IADL performance, might be able to provide a more sophisticated biomarker candidate. Thus, the present study aimed to adopt EEG combined with the Amsterdam Instrument of Daily Living Questionnaire‐Short Version Traditional Chinese (A‐IADL‐SQ‐TC) to early detect cognitive decline in the preclinical stages.

**Method:**

We recruited 76 participants, including those with normal cognition (n=25), SCD (n=21), MCI (n=17), and dementia (n=13) in both community and memory clinics. All of the participants were measured with the Mini‐Mental State Examination (MMSE), Montreal Cognitive Assessment (MoCA), and eye‐closed EEG recording. Furthermore, all proxies of the participants filled out the A‐IADL‐SQ‐TC.

**Result:**

Individuals with MCI and dementia exhibited larger relative power in the low‐frequency bands, specifically the delta (0.5‐4 Hz) and theta bands (4‐8 Hz), and smaller relative power in the high‐frequency band, specifically the alpha (8‐13 Hz) and beta bands (13‐20Hz). These findings are consistent with previous research. Additionally, we discovered that the A‐IDAL‐Q‐SV‐TC T‐score negatively correlated with theta relative power across the scalp (r = ‐.55 to ‐.62, all p**<.001), while positively correlated with beta relative power across the scalp (r =.35 to ‐.46, all p<**.001). Regarding the global theta relative power, the one‐way ANOVA showed significant group differences, F (3,72) = 19.768, p<.001. However, the post‐hoc effect revealed only the dementia group showed a significantly higher relative power than the other groups.

**Conclusion:**

We have discovered that the resting‐state EEG oscillations exhibited different patterns across different stages of dementia. Furthermore, A‐IADL‐SQ‐TC was associated with the spectral intensity of the resting‐state EEG oscillations, and together they provide more sensitive indicators for early detection.